# Voice, (inter-)subjectivity, and real time recurrent interaction

**DOI:** 10.3389/fpsyg.2014.00760

**Published:** 2014-07-18

**Authors:** Fred Cummins

**Affiliations:** UCD School of Computer Science and Informatics, University College DublinDublin, Ireland

**Keywords:** joint speech, participatory sense-making, intersubjectivity, dynamic entwining, chant

## Abstract

Received approaches to a unified phenomenon called “language” are firmly committed to a Cartesian view of distinct unobservable minds. Questioning this commitment leads us to recognize that the boundaries conventionally separating the linguistic from the non-linguistic can appear arbitrary, omitting much that is regularly present during vocal communication. The thesis is put forward that uttering, or voicing, is a much older phenomenon than the formal structures studied by the linguist, and that the voice has found elaborations and codifications in other domains too, such as in systems of ritual and rite. Voice, it is suggested, necessarily gives rise to a temporally bound subjectivity, whether it is in inner speech (Descartes' “cogito”), in conversation, or in the synchronized utterances of collective speech found in prayer, protest, and sports arenas world wide. The notion of a fleeting subjective pole tied to dynamically entwined participants who exert reciprocal influence upon each other in real time provides an insightful way to understand notions of common ground, or socially shared cognition. It suggests that the remarkable capacity to construct a shared world that is so characteristic of *Homo sapiens* may be grounded in this ability to become dynamically entangled as seen, e.g., in the centrality of joint attention in human interaction. Empirical evidence of dynamic entanglement in joint speaking is found in behavioral and neuroimaging studies. A convergent theoretical vocabulary is now available in the concept of participatory sense-making, leading to the development of a rich scientific agenda liberated from a stifling metaphysics that obscures, rather than illuminates, the means by which we come to inhabit a shared world.

## 1. Introduction

We speak with confidence of something called “language,” as if this term referred to a single system, capable of multiple forms of manifestation (writing, speech, signing), but unified by organized structures and processes in the formal domains of phonology, morphology, syntax, and semantics. This emphasis on systematicity and symbolic encoding has utterly dominated the scientific view of “language” at least since the structuralist innovations of Saussure (1959/1916), and has been greatly reinforced by the pivotal role of generative linguistics in the birth of the cognitivist account of mind as a form of symbol-based information processing (Fodor, 1975). In the context of inter-personal communication, language, on this view, serves as a form of message passing, whereby ideas conceived in the mind of one person are encoded, first into words, and then into movements of mouth or hand, at which point they become transmittable to another, who sets about decoding them, thereby gaining access to the ideas of the sender. The message passing perspective on language is compelling, powerful, and supported by a host of technologies, from the very first forms of writing to the most sophisticated of digital platforms.

The emphasis on symbols and systematicity allows the identification of a tentative boundary between the linguistic and the non-linguistic. For example, a conventional distinction is drawn between phonological and non-phonological characteristics of the sounds of speech. Roughly, those features that support the identification of discrete categories such as phonemes, are taken as indices of linguistic structure, while non-categorical and continuously varying features such as the loudness of a voice would lie beyond the notional bounds of language proper. Once discrete entities belonging to non-overlapping categories are available, they can be combined into larger symbolic structures, from syllables to novels.

Language thus appears to be a clearly delineated and unified phenomenon, of which one can meaningfully construct theories. This leads to a compelling observation that there seems to be a yawning chasm between the many kinds of communication systems found in animals and the generative, creative richness found in every human language. And so the foundations are laid for the perplexing observation that language seems to have appeared not so very long ago in an evolutionary timescale, and to have immediately enabled the development of the whole of human culture, technology, and all the institutions of all societies.

Two related observations will serve to provide us here with a slightly different view of “language.” The first is that *the above story is fundamentally committed to an ontological split between mind and world*. If we accept such a split, then meanings or ideas belong firmly in the realm of the mental, and they find expression indifferently in writing or speech, each of which provides a kind of physical container for the passing of ideas from one mind to the next. The second observation is that *the traditional story enforces a somewhat arbitrary divide between the linguistic and non-linguistic*, motivated by the desire to ensure that language is systematic and supports the kind of symbolic operations familiar from syntax and related disciplines. If we observe communication among people, we see many aspects to that behavior that never feature in linguistic theory, and that nevertheless seem to be reliably and essentially associated with inter-personal communication. These two observations are related, because if we consider alternatives to the Cartesian mind/world split that divides ideas and meanings from sounds and movements, the apparent significance of many of the behaviors and features reliably and regularly attending communication may change, and with that, the boundaries of “language” may shift, or, indeed, fragment, to reveal a variety of phenomena that do not admit of a single systematic description.

I will argue that the way in which we conventionally treat of the phenomenon called “language” is overly restrictive, and seems more appropriate to the characterization of writing than speaking/listening (Linell, 2005). Older than writing by far is the voice, and the voice has remarkable properties all of its own. Chief among these is the obligatory association between the voice and a transient subject-pole that grounds intentionality. This, it seems to me, may be part of the reason the inner voice seems to be inextricably associated with the Cartesian subject. To develop this notion, I will turn to the substantive domain of joint, or collective, speaking, showing how collective speech engenders a different kind of subject, displaying collective intentionality. Furthermore, just as the voice of the individual admitted of development and codification in writing, so collective speaking admitted of development and codification in practices of liturgy and ritual. Written language, which is the more accurate target of modern linguistics, is thus not the only descendent of voice. The empirical study of collective speaking is in its infancy, but it reveals emergent phenomena that arise only in the real time reciprocal interaction of speakers speaking in unison. These emergent phenomena add substance to the argument that the traditional depiction of language as message passing mischaracterizes, or omits, much of what is going on in vocal communication (Cowley and Love, 2006). It neglects the fluid intertwining of subjectivities that arises in real time reciprocal interaction, and that appears clearly in joint speaking. This only becomes apparent if we approach languaging (rather than language) as a set of multi-faceted behaviors that defy characterization from a single metaphysical viewpoint^1^.

## 2. Revisiting descartes

Let us fancifully drop in on Descartes as he deduces his own existence. The statement “Cogito, ergo sum” is without doubt the most famous line in Western Philosophy, and the basic outline of the argument underlying it is overly familiar^2^. A skeptical philosopher, wishing to establish a foundation for true and certain knowledge, recognizes that the world of appearances, mediated by the senses may be illusory. He considers what remains after denying the testimony of the senses, and reasons thus:

So after considering everything very thoroughly, I must finally conclude that this proposition, *I am, I exist*, is necessarily true whenever it is put forward by me or conceived in my mind (Meditation 2, AT 7:25).

The “I” that is invoked here is explicitly and emphatically not a body, but a mind (7:27). The split between mind and world is absolute. Irrespective of how the consequences are played out, Descartes' certainty has become the split we have failed to distance our selves from. Substance dualism narrowly conceived is, of course, not a respectable metaphysical position any more, but the split that is effected here between mind and world, and at the same time, between metaphysics and epistemology, far from being overcome, has become the foundational assumption upon which the whole of psychology (and more) has been built. As Sheets-Johnstone put it, it has become “a lexical band-aid covering a 350-year-old wound generated and kept suppurating by a schizoid metaphysics” (Sheets-Johnstone, 1999, p. 275).

But what is going on for Descartes? There is a voice. Whether it is a voice speaking in Latin “Cogito, ergo sum!,” or a voice speaking in French “Je suis, j'existe!,” it is a (silent) utterance—a thought in the form of words. Without language (better: languaging), there is no such thought. Without a culturally specific history of vocal interaction among people during which meanings and uses of language emerge, there is no such voice. The solipsistic prison of Descartes' fancy is not so devoid of other people as he seems to believe, for in harboring the voice that can utter the “Cogito!,” it is populated by the practice of Latin, or the practice of French. Closing the eyes does not keep out the world, and it does not keep out other people.

The inner voice of linguistic thought that speaks here “to” Descartes is not different in kind from the outer voice of overt speech. Indeed, the whole metaphorical quagmire associated with the use of the terms *inner* and *outer* stems from the very confusion I wish to here circumvent. Vygotsky has presented a thorough argument that the overt but self-directed speech of young children is, firstly, a specialization of intersubjective social speech, and secondly, is the precursor to inner speech, or linguistic thought (Vygotsky, 1986). This insight provides us with an understanding of continuity between overt speech and silent speech, or linguistic thought.

What if we choose to interpret Descartes' predicament somewhat differently? Instead of considering the voice as evidence of a pre-existing subject, we might consider it to *give rise to* a transient subjecthood. We cannot understand the occurrent thought as an utterance in the message-passing sense, as there are not two distinct domains, a speaker and a listener, for any message to be passed among. But we are now entertaining the tentative notion that there is no Cartesian subject before the occurrence of the thought, and so any subjecthood associated with this utterance arises with the utterance and fades thereafter. This is not a fully fledged psychological subject, equipped with the mechanisms of “cognitive systems”; it is a subject-pole that allows a distinction between subject and world, or self and other, to be discerned, and that supports or invites the ascription of intentionality. It is a transient orientation, tied to the real time unfolding of the linguistic thought itself (“*… **whenever** it is put forward by me or conceived in my mind*.”). Later in the 2nd Meditation, Descartes himself seems to concur with this association of the Subject with the transient inner voice when he says “I am, I exist—that is certain. But for how long? For as long as I am thinking. For it could be that were I totally to cease from thinking, I should totally cease to exist” (Meditation 2, AT 7:27). Now the nature of “thinking” has not been generaly agreed upon, but the form of thinking Descartes here alludes to is clearly the utterance of an inner voice, in specific words, words which he is capable of repeating to us, words which we can characterize as Latin or French. I wish to pursue this idea, that *voice* gives rise to the complementarity between the poles of subject and world, and it does so in real time.

## 3. Voices and subjects

[V]oice is a kind of sound of an ensouled thing. For none of the things without soul gives voice, though some are said by analogy to give voice, such as the flute and the lyre and whatever other of the things without soul have the production of sustained, varied and articulate sound. For voice also has these features and so there is a likeness (Aristotle, 1986 420b, p. 178).

The association between the animate (even ensouled) subject and the voice is ancient. In Connor (2000) the long history of the subjects perceived as being behind voices emanating from unlikely places is recounted in detail. From the Delphic oracle through the medieval fascination with demonic possession, prophecy, and divine inspiration, voices perceived as coming from the stomach, the genitals, or even a crack in the rock have been enthusiastically attributed to invisible subjects, rather than to sound-producing properties of either inanimate objects or of atypical parts of the body itself. Much of the ghoulish fascination that the ventriloquist's dummy attracts lies in the obligatory projection of a subject behind the grotesque appearance. Connor writes:

For I *produce* my voice in a way that I do not produce these other attributes [eyes, hair, gait, fingerprints, etc]. … giving voice is the process which simultaneously produces articulate sound, and produces myself, as a self-producing being (Connor, 2000, p. 3).

It is telling that the words uttered in one of the very earliest sound recordings, made by Alexander Graham Bell in 1881, are “T-r-r—T-r-r—There are more things in heaven and earth Horatio, than are dreamed of in our philosophy—T-r-r—*I am a Graphophone* and my mother was a Phonograph” (Volta Laboratory, 2013, Emphasis added), thus instinctively investing one of the very first disembodied voices born of technology with subjecthood of its own. Remarkably, the telephone and the phonograph came into being almost simultaneously—in 1876, 1877. Add to these the advent of radio transmission of the human voice, first done in 1900 in Brazil, and it is clear that we have been awash in disembodied voices for over a 100 years and counting. The irritating proliferation of pseudo-personalities such as the iPhone's Siri seems likely to continue.

If the voice Descartes conjures up alone generates a subjectivity that is aligned with the classic subject-object distinction at the level of the single individual, then we might give consideration to the possibility that voice employed in different circumstances might generate other forms of subjectivity, without commitment to individual Cartesian minds.

### 3.1 Shared subjectivity and common ground

When an utterance is made in a specific context with speaker and listener both present, it is interpreted in the light of the shared understanding of all parties. This has found expression in theoretical notions of common ground (Clark and Brennan, 1991), or socially shared cognition (Schegloff, 1991). Most developments of the idea of common ground are couched within the information processing/message passing framework, and therefore make use of some version of aligned or shared representational content. However, it is not necessary to appeal to such unobservable constructs from a hidden Cartesian world (Hutto and Myin, 2013). There is ample evidence that participants in a conversational exchange become mutually linked in many subtle but observable ways. Eye movements (Richardson et al., 2007), postural sway (Shockley et al., 2009), and even blinking (Cummins, 2012) have all been found to become subtly intertwined in conversation, leading to a dynamic entanglement of the participants. Speakers and listeners are further linked through the provision by the latter of signals of ongoing engagement through postural, gestural, and vocal indices or backchannels (Wagner et al., 2014).

The yoking together of two or more people engaging in language behavior establishes a common basis from which the participants confront the world. It makes available a shared framework within which statements can be interpreted. It thus provides a scaffold for shared intentionality (Carr, 1987). The ability to share an intentional perspecitive seems to be at the very heart of human language use, but it is not an all or nothing affair. Two protesters with common purpose who chant the same slogan demonstrate an extreme alignment with respect to the world. But two people engaged in heated disagreement must still achieve a great deal of alignment in order to disagree felicitously. The topic of disagreement must be foregrounded, at the expense of everything else. In disputing causal chains, in laying out competing sequences of events, and in presenting different interpretations of the significance of actions and events, two disputants are necessarily sharing a great deal of background framing, picking out these events rather than those, identifying the same actors, while quarreling over their respective roles. Even in the absence of conversational exchange, people observing the same scene exert reciprocal influence on one another, such that their gaze behavior, and by inference, the details they pay attention to, become inter-dependent. In a series of experiments summarized in Dale et al. (2013) gaze behavior of subjects are demonstrated to depend sensitively on the presence of others, and on whether one subject knows or believes that the others are seeing and hearing the same things as they are.

If joint languaging provides a very powerful example of intentional alignment, then it might be that that the ability to coordinate the manner in which we jointly pay attention to the world is an important skill that facilitated the emergence of such behavior, as argued in Fusaroli and Tylén (2012). Sometime between the last speciation event some 5 or 6 million years ago that gave rise to chimpanzees and bonobos on the one hand, and the hominid line on the other, something happened that had profound consequences for our ability to share perspectives and to coordinate with one another. There is one small biological change that we know occurred in that time, that might play a significant role here. That change gave rise to the white sclera of the human eye that contrasts vividly with the darker iris, thus providing a very clean signal of the direction of gaze of a partner (Tomasello et al., 2007). The other great apes do not have such a contrast, and their ability to align their gaze is severely limited, and based on head direction rather than the eyes—although chimpanzees and bonobos in particular do display some evidence of understanding the visual perspective of another (Okamoto-Barth et al., 2007). The ability to follow each other's gaze thus facilitates the sharing of attention, and has been demonstrated to structure mother-child interactions, while inducing the abilty to take part in languaging (Tomasello and Farrar, 1986).

As common ground is established, the subjective point from which utterances are spoken also shifts. Vygotsky has pointed out how the (linguistic) subject becomes an implied, rather than an overt, element in speech once common understanding has been established (Vygotsky, 1986, p. 236). For example, it would be odd to respond to the question “Would you like a cup of tea?” with the answer “No, I don't want a cup of tea,” instead of simply “No.” Similarly, a group of people waiting for a bus establishes sufficient shared context that no one is likely to point out the obvious and say “The bus for which we are waiting is coming,” but simply “coming” or some such expression. The dropping of the linguistic subject is more extensive yet in inner speech, of which Vygotsky says “it is as much a law of inner speech to omit subjects as it is a law of written speech to contain both subjects and predicates” (Vygotsky, 1986, p. 243). Many languages allow dropping of any explicit mention of the subject once they can be inferred on pragmatic grounds. This is not merely a syntactic quirk of one group of languages, as it is found in such typologically distant languages as Japanese, Chinese, Turkish, and Spanish (Huang, 1984).

It would be a mistake to simply equate the subject pole of a subject-world complementary pair with the syntactic subject, but it would be inexcusable too to ignore the deep link between the fundamental linguistic structure of subject and predicate on the one hand and the subjective pole from which utterances are brought forth on the other. The subject pole that arises in the unfolding of the voice grounds intentionality, and provides an anchoring point for reference. This is, perhaps, most explicit in the manner in which deixis functions, allowing use of terms such as “there,” “here,” “then,” “now,” whose meaning is anchored in the joint situation created by conversational participants; It is also explicit in the manner in which the first personal pronouns, both singular and plural, find flexible, and context-specific use. It is implicit too in establishing a shared register and perspective within which meaning is negotiated. The differentiation of subject and world, and the ability to establish a shared perspective within which utterances function, precedes any overt syntactic knowledge or awareness by millennia (Olson, 1996).

### 3.2 Alignment vs. synergy

The dynamic intertwining of conversational participants interacting in real time has not gone unnoticed. An influential approach to account for the many overt and subtle ways in which two interlocutors become linked is found in the Interactive Alignment model of Pickering and Garrod (2004, 2014). This model seeks to describe the tendency for conversational partners to imitate one another at a variety of levels, from syntactic biasing, through lexical selection, down to the level of phonetic, and gestural imitation. The idea that similarity in one domain can unconsciously bleed through representational levels to generate similarity in other domains provides some explanatory purchase on a great deal of corpus-based data. As a general account of the dynamic coupling and mutual accommodation found among speaker/listeners, however, it is somewhat limited. It leaves language resolutely within the heads of individual conversing partners, and this does not move beyond the Cartesian, representationalist framework. It is “representation-hungry,” demanding computational representations at many levels, and indeed, in its most recent form, it conjures up a baroque series of simulations inside the heads of individuals who must not only act, but also predict the actions of others (Pickering and Garrod, 2014). This approach does not generalize in any obvious way to multi-party conversations. Nor does it account for coupling among interactants that are not strictly imitative in nature, as with the mutual influence exerted on blinks (Cummins, 2012). The tendency to alignment suggests that felicitous conversation would result in mere mimicry, which is again not what we observe, and it privileges similarity, at the expense of complementarity, thereby missing the fundamental role-based nature of conversation in which the positions of speaker and listener alternate.

A competing account has recently been proposed that regards inter-personal coordination in dialog as a form of synergy or dynamical coupling (Fusaroli et al., 2014). This approach is rooted in dynamical approaches to coordination that are level-agnostic, seeking to understand emergent phenomena at one level (e.g., the dyad) as arising through processes of self-organization from the constrained interaction of autonomous components at a lower level (the speaker/listeners) (Kelso, 1995; Latash, 2008). This approach highlights the sensitivity of participants to real time recurrent interaction, as is evident even in the early interactions of infants and mothers (Murray and Trevarthen, 1986). It emphasizes the intertwining of the movements of participants, leading to dimensional reduction, so that two interacting persons become, temporarily, a simpler collective entity than the two persons considered as a mere conjunction of individuals. It acknowledges both synchronized and complementary actions as they contribute to this simplification, and it emphasizes the manner in which shared understanding of task constraints leads to stability of patterning in time. Although still somewhat speculative, this level-independent approach seems commensurate with the approach to be developed here that treats groups of people as synergetically organized domains in their own right, with respect to which subjectivities of a collective nature can be identified.

Synergistic approaches to human communication have been argued for by others. Thibault (2011) adopts a position not unlike the present one in which a fundamental distinction is drawn between what he calls talk and text. The role of voice described both here and in his work emphasizes the bodily entrainment that arises at a very fine scale among interactants, while the properties that linguists conventionally consider, and that admit of a computational description, constitute a distinct, and second-order set of phenomena. Although not focussed on languaging, Riley et al. (2011) argue that interpersonal movement coordination is the result of establishing interpersonal synergies of the sort described here, and they distinguish between component-dominant dynamics, as portrayed within a cognitivist framework, with interaction-dominant dynamics in which the autonomy of the level of interaction is more thoroughly acknowledged. Finally, the perceptual crossing paradigm introduced by Auvray et al. (2009) provides a minimalist experimental set up in which two people interact in real time in a minimal virtual space. While not communicative in any conventional sense, the nature of the emergent behavior observed serves to illustrate the principal point being made that the interaction itself constitutes a level of relative autonomy that is not reducible to the conjunction of properties of its components (Froese et al., 2014). These latter two examples illustrate that social interaction and languaging are not separate phenomena. Languaging is a constitutive part of the manner in which interpersonal entrainment or coupling arises in the moment by moment real time reciprocal interaction among people.

### 3.3 Voice vs. writing

Before giving further consideration to the relationship between subjecthood and voice, it is appropriate to recall the vast chasm that separates speech from writing, not least as the claim is made here that most of the phenomena described by modern linguistics relate, in fact, to the structure of written communication, and are only indirectly relevant to the act of speaking, which is the central form in which languaging is manifested (Linell, 2005). Since the advent of alphabetic writing in Greek society, a naive view has been available that writing is simply a device for transcribing speech. Olson (1996, p. 66) identifies overt statements that express this view from Aristotle, Saussure, Bloomfield, and more. This is why theories of syntax, morphology, and semantics, that together delimit much of that which we call “language,” allow themselves to study and model the formal characteristics of symbol strings, without consideration of the medium of expression. This insensitivity to the enormous differences between writing and speech underlies the focus by Saussure on *langue* rather than *parole*, and by Chomsky on competence, rather than performance. With that, modern linguistic theory has turned its attention away from the most common form of languaging, indeed the only one that existed from the fuzzy origins of speech until the relatively recent development of writing and the even more novel phenomenon of mass textual proliferation. It has ignored the real time reciprocal interaction among people giving voice from context-specific situations of concern.

We have now a wealth of research that documents very substantial changes that arise with the advent of writing, and especially with the spread of literacy consequent to the development of printing. These changes affect not only the way language is used, but the very structure of the consciousness of language users (Stewart, 2010). Ong (1982) provides an authoritative and comprehensive catalog of differences between the way knowledge is managed, shared, and verbalized in primary oral cultures, and in highly literate ones. Olson (1996) further documents the profound conceptual and cognitive implications of the spread of literacy. Much of this work focusses on the novelties that accompany writing and literacy. McLuhan claimed that “writing was an embalming process that froze language” (McLuhan, 1964), and he provides an anectode from Prince Modupe, who speaks of his encounter with the written word in his West African days:

The one crowded space in Father Perry's house was his bookshelves. I gradually came to understand that the marks on the pages were *trapped words*. Anyone could learn to decipher the symbols and turn the trapped words loose again into speech. The ink of the print trapped the thoughts; they could no more get away than a *doomboo* could get out of a pit… (McLuhan, 1964, p. 84).

With writing, texts achieve an independence from their sources. A spoken utterance is necessarily vouched for by the speaker, while a written sentence asserts, without the contingency and commitment of a speaker. I have mentioned that voice gives rise to a subjective pole. Here we can see that the complement is also true: writing gives rise to a particular kind of *objectivity*, one in which for the first time it is possible to have “facts that speak for themselves” (Latour, 2013). (For an insightful account of several ways in which objectivities are constructed, see Daston and Galison, 2007). Written sentences remain immutable and thus support dissection and analysis in a way that spoken utterances, which must be articulated each time they come into being, do not. The further development of speech and language technologies in the service of message passing has given rise to forms of spoken langauge, e.g., in news broadcasts or public service announcements, that bear greater similarity to written texts than to spoken utterances, while recent increases in the possibility of text-based reciprocal exchanges, e.g., in SMS messaging, further serve to complicate the relation between voices, texts, messages, and intentions^3^.

It is interesting in this regard to consider the constraint observed by Everett to hold in the language of the Pirah~a, an Amazonian tribe whose language is remarkable in its simplicity and omissions, having no counting system, very restricted tenses, arguably no syntactic recursion, etc. The Pirah~a also have no mythology or stock of fiction. Everett attributes many of these constraints to what he calls the Immediacy of Experience Principle, according to which statements by the Pirah~a “contain only assertions related directly to the moment of speech, either experienced by the speaker or witnessed by someone alive during the lifetime of the speaker” (Everett, 2009a, p. 132). Here, the strong tie betwen the speaker and the words spoken appears to have become sedimented into the very structure of the language and culture, leaving no room for the disembodied words found in writing. It is perhaps no coincidence that Everett's observations have become controversial precisely among those linguists who hold syntax, and syntactic recursion in particular, to be central to the very nature of language (Hauser et al., 2002; Everett, 2009b).

## 4. Speaking in unison

The act of speaking in unison is a common form of vocal behavior that is accorded no particular theoretical significance in a message passing view of language. On the received view, minds and subjects are closed and singular; thus many people saying the same thing at the same time appears merely as a multiplication of the individual speaker. The behavior does seem somewhat perplexing though, for what message is being passed if we all know the words? It is worthwhile to consider both the occasions in which people often speak in unison, and the form of the speech so produced.

“Joint speaking” is an umbrella term I have coined to cover all occasions in which the same words are uttered by multiple people in unison (Cummins, 2013a). This includes many practices of collective prayer, the chants of both protest demonstrators and sports fans, the recitations of young school children, performances of choral speech, and the swearing of collective oaths in secular contexts. To all these naturally occurring variants we can also add the simultaneous reading of novel texts by pairs (or more) of speakers in the laboratory in a paradigm known as Synchronous Speech (Cummins, 2003, 2009).

This brief survey of situations in which people speak in unison makes it clear that this behavior is very widespread, and is found in virtually every culture. It is thus a central, and not a peripheral, example of languaging. With the exception of joint speaking in classrooms, which serves a multitude of purposes imposed by educational authorities rather than expressing any sentiment of the speakers, all of the naturally occurring forms of joint speech are found in situations in which the attribution of collective, shared, intentionality seems to straightforwardly capture the significance of the practice for participants. In prayer contexts, collective speaking testifies to shared beliefs. In protest, the shared purposes of the crowd are made manifest through chanting. Among sports fans, chants are a means by which collective identity is sustained and asserted. None of this is at all surprising, nor in need of precise definition—at least, no more precise than seems warranted for the attribution of beliefs, desires, and intentions to individuals. While we may not all be enthusiastic chanters, even a reluctance to join in such behavior testifies to the obligatory assocation of such voicings with the underlying sentiments.

But if message passing does not illuminate such behavior, it seems fair to ask how we might better characterize it; why are people engaging in such vocal activity, if not to pass ideas around? While there is probably not a single answer to this question, a useful conceptual approach suggests itself from the theory of speech acts (Austin, 1975). Austin noted that many utterances achieve something simply by virtue of being spoken. Examples include “I pronounce you man and wife,” or “I apologize for my behavior.” Such utterances he called “performatives.” In the treatment provided by Austin, they are frequently signaled by such verbs as “pronounce,” “decree,” “promise,” etc. The set of performatives Austin alludes to, and the associated set of acts performed is very restricted. If there is merit to the idea that uttering gives rise to the complementary poles of subject and world, then *all* utterances might properly be considered to be performatives, and the establishment of a transient subject pole with an implicit intentional structure would then be an achievement of the act of uttering. This approach to understanding joint speech helps to make sense of some of its most reliable features. In what follows I will consider mainly the three most common forms of joint speech^4^: collective prayer, protest chanting, and sports chanting.

All three forms of joint speech are frequently, almost inevitably, characterized by repetition: the same phrase or short verse is repeated tens, or even hundreds of times over. Repetition makes sense if the temporally bound act of utterance is required to establish and maintain a transient subject pole with respect to which we can identify beliefs or intentions. Repetition is undergirded by physical actions such as fist pumping, bead twiddling, or arm waving. While bead manipulation is relatively private, the more macroscopic actions further serve to facilitate synchronization among participants.

Repetition also serves to accentuate and exaggerate the rhythmic properties of utterances, while repetition of a short phrase can also induce a change in perception from speech to song (Deutsch et al., 2011). In repeated spoken chants, the form of speech that arises thus blends seamlessly into the musical domain, establishing a continuity between speech and music. The close relation between spoken and sung chant is signaled by the very ambiguous nature of the word “chant” in English which applies with equal facility in either domain. It is interesting that a focus on collective speech makes a continuum between speech and music appear natural, even obligatory, while the message passing perspective as articulated most clearly by Pinker (1999) insists on an absolute divide between the two domains. On the message-passing view, speech is an expression of the highly valued notional faculty of language, and thus central to our human minds, while music is denigrated as “auditory cheesecake,” with no—from his perspective—apparent functional significance, thus meriting being grouped together with artistic expression, cheesecake, and pornography (Pinker, 1999). If anything illustrates the limited capacity to describe, or even see, that the message passing perspective induces, surely it is this failure to appreciate the continuum we are all familiar with that extends from instrumental music, through song, rap, poetry, rhymes, rhetoric, and chant (Cummins, 2013a). We might note in passing that the contrast between the real time participatory nature of the voice that is here contrasted strongly with the frozen nature of writing finds a strong parallel in contemporary discussion of the relationship between live musical performance and recording (Chanan, 1995).

We like to speak of the “wisdom of crowds,” but the rather more familiar notion of the ignorance of the mob, whose powers of reason are not to be trusted, is perhaps more apt for many of the situations under consideration. While groups have frequently been found to outperform individuals in tasks of judgment and estimation (Koriat, 2012), groups involved in joint speech of protest are often found in volatile situations where collective actions are rudimentary and aggressive. It is worth noting though that some degree of sophistication in the beliefs that are jointly articulated is provided by the formal scaffold of call and response. The device of having a single leader call a series of questions to which the crowd provides a series of responses is found in both prayer and protest, though perhaps less so in sports chants. In prayer, this sequence of leading call and collective response is often formalized into liturgical rites, allowing for a great deal of complexity in the beliefs that are thereby expressed. In protest, it is far more common to see only a single call, and a single response, and the very nature of protest mitigates against the kind of codification found in ritual liturgical practices. Sports chanting seems to be more concerned with the demonstration of collective identity than with the formulation of explicit statements of belief or intention, and call-and-response chants are less common.

If we view writing as an elaboration of some aspects of speaking, i.e., a technological extrapolation that gives rise to a formal system of the kind studied under the somewhat misleading label of “language,” then we might observe that vocal behavior, or languaging, appears to have other extrapolations, other forms of extension, and other forms of codification, so that the formal constructs of the linguists are not the only descendents of the voice. Collective speech has found integration into rituals in a great diversity of traditions. The Abrahamic religions all formalize collective speaking within their respective services, and in each of them the rituals integrate joint speaking into a carefully orchestrated sequence of complementary acts by service leaders and participants that include highly stylized sequences of movements such as bowing, kneeling, marching, etc. Other religious traditions have engaged in similar forms of codification (Bell, 1988). Parallels between linguistic grammar and ritual structure have previously been noted (Michaels et al., 2010), but the principal point argued here is that voice has given rise to more than one species of formalization. Liturgy and ritual do not admit of the same generative mutability as freely spoken or written text, but by codifying such utterances in collective speech and ritual, the implicit intentional structure that arises in speaking and performing, together with the associated belief structure, is stabilized. With such observations, the boundaries of “language” become somewhat less determinate, and the subjects that find voice become both more numerous and more varied.

### 4.1 Dynamic entanglement in synchronous speaking

If the relation between voice and subjectivity put forward here has merit, joint speaking appears as an extreme example that can serve to hone our considerations of the form and nature of collective intentionality. In monolog, I alone dictate the intentional ground of my utterances; in conversation, the shared ground is fluid and negotiated; in chanting it is immovable. Are there then any signatures of joint intentionality that we can observe? In the spirit of the dynamical coupling hypothesis of Fusaroli et al. (2014), we might look for evidence that joint speakers are strongly coupled, giving rise to emergent phenomena at the supra-individual level.

In a series of behavioral studies in which speakers are asked to read novel texts in unison, no major differences that would serve to pick out speech as collective based on its acoustic characteristics alone have been observed (Cummins, 2014). Speech produced in these constrained laboratory settings is remarkably unremarkable, and the technique of having subjects speak in synchrony has been used as a device for obtaining unmarked speech in several phonetic studies (Krivokapić, 2007; Kim and Nam, 2008; O'Dell et al., 2010; Dellwo and Friedrichs, 2012). The unmarked phonetic structure of speech elicited in the synchronous speaking situation contrasts strongly with the observation that texts recited in ritual and rite are frequently, if not inevitably, highly stylized in prosodic form. For example, consider the typical pattern with which the Hail Mary is said when reciting the rosary, or, in a secular context, the characteristic form of the Pledge of Allegiance as recited by American schoolchildren. Prosodic stylization thus appears as a reliable, but not necessary characteristic of joint speech.

There is one form of speech error found in a synchronous speech task that seems to be unique to that situation, and that illustrates a strong dynamic coupling between speakers. When one speaker makes a speech error, it is frequently, though by no means always, observed that both speakers stop speaking simultaneously. Sometimes this abrupt cessation can even be in mid-syllable. Abrupt and simultaneous cessation of speech seems to be unique to this situation, and I have previously compared it to the collective tumbling that happens so readily in a three-legged race if either participant makes a misstep (Cummins et al., 2013). This seems to suggest that the task of synchronizing leads to a close intertwining of the process of speech production by each speaker, leaving each vulnerable to mistakes by the other. This observation might be tempered, however, by noting that the degree of synchronization found in the laboratory is typically much greater than that found in the wild, where relatively loose temporal alignment is common and tolerated.

A second source of empirical phenomena associated with joint speaking comes from an fMRI study by jasmin et al. (in preparation), in which subjects spoke prepared sentences in a variety of conditions, including speaking alone, listening, speaking in synchrony with the experimenter and speaking in synchrony with a recording of the experimenter. Importantly, subjects were not informed of the difference between the latter two conditions, and on debriefing, they were never aware that recordings were used at all. In contrasting the regional blood flow subsequent to speaking in the latter two synchronization conditions, a marked difference was found in macroscopic patterns of cortical activity, despite the obliviousness of subjects to the contrast. In particular, synchronization with a live person was characterized by an increase in activity in right hemisphere locations, including the temporal pole, supramarginal gyrus, superior temporal gyrus, and the right hemisphere homolog of Broca's area—the latter three are areas that, in the left hemisphere, are reliably implicated in speech production activity. There is thus a large scale alteration to the well-known hemispheric asymmetry that attends speech production, but only when the speaker is coupled in real time to another speaker, and not when the non-self voice has the inflexibility of a recording.

## 5. Voice, (inter-)subjectivity and real time recurrent interaction

As scientists, there is a need to acknowledge that the metaphysical background within which one works makes some inquiries possible, and some impossible. For all the acknowledged successes of the message passing view of language rooted in a Cartesian framework, there are very many familiar phenomena that have been passed over, or, at best, relegated to the outer wastelands of the non-cognitive and non-linguistic. I have here sought to work with a notion of the subject that is an emergent property of specific kinds of interpersonal interaction rooted in real time reciprocal exchange. This unconventional view of the subject brings with it a very different view of what language is, to the point where the systematic formal system described by modern linguistics no longer appears to be describing the human capacity to create shared perspective, to generate a shared common ground, and to bring forth a common world. Where received approaches to “language” treat of regularities found in sequences of symbols, I have focussed on the voice, uttered from a specific concerned perspective, and necessarily tied to the real time negotiation of a subjective pole. In the voice, we find a strong index of intentionality, but an intentionality that shifts, that arises fluidly, that is sometimes grounded in an individual, sometimes in a negotiated context, and that sometimes seems to emerge at the collective level in a manner no longer reducible to the thoughts, beliefs, and perspectives of the contributing individuals (Carr, 1987). This dissociation of the voiced subject from the solipsistic individual is seen perhaps most clearly in the case of joint speech. The emphasis on voice and intentionality serves to position the symbolic domain of structural and generative linguistics as a specific, limited, extrapolation, and codification of an older practice of *uttering* that has given rise to several distinct extensions and codifications in such domains as ritual and rite.

The loosening of metaphysical commitments that results when we abandon the Cartesian subject offers the opportunity to reconsider many phenomena, and joint speech provides an important and familiar case in point. The practice of joint speech is not restricted to any particular culture. As well as being ubiquitous, it is immediately apparant that the situations in which people speak collectively do not form an arbitrary or incoherent set. All such situations seem to provide strong evidence of collectively held beliefs, and it is through the collective voicing that this attribution becomes warranted. It might help here to note that the subjectivity being treated so rudely is not coextensive with the mind of an individual, nor with the idea of a cognitive system, conceived of as a set of sub-personal information processing mechanisms that some hypothesize to underlie observed behavior. The subject pole referred to here is an aggregate to whom it makes sense to attribute a limited range of intentions, and in particular, beliefs. I am thus wielding the term “belief” here in a sense rather like the dispositional account provided by Ryle (1949). This flexible notion of the subject seems to work when applied to an individual, a conversing dyad, or a lynch mob, each of whom can be said to speak from a distinct position, with a specific perspective. In strenuously avoiding the Cartesian split between mind and world, we would do well to avoid adopting an overly rigid metaphysical position. Rather, if subjects admit of the kind of treatment proposed here, then an ontological lightness of touch that can encompass many kinds of intentional subjects seems warranted.

The empirical phenomena described above strongly highlight the importance of real time dynamic interaction among people in generating the subject-pole to which beliefs can sensibly be attributed. The neural signature of collective speaking is found when speaking with a live speaker, but not with a recording (jasmin et al., in preparation). Live conversational partners become entangled not only in ways that fit a linguistic description (lexical priming, syntactic biasing, phonological, and phonetic imitation, Pickering and Garrod, 2004), but in a host of subtle ways that have hitherto been treated of as non-linguistic. These include gaze, posture, gestures, and blinks, but this set might conceivably be considerably extended as researchers turn their attention more and more to physiological markers of interaction (Campbell, 2007; Richardson et al., 2007; Shockley et al., 2009; Cummins, 2012; Wagner et al., 2014). The voice is an important part of the means by which a collective perspective is established and maintained, but it is one among many. The interaction of voice and gaze may play a particularly strong role in allowing the protracted sustainment of conditions of joint attention, which appears as a possible foundation for the shared intentionality required to ground a human cultural world (Tomasello et al., 2005)^5^.

The dynamic entanglement seen in conversation, and in joint speech, can be empirically described as a form of mutual coordination, whereby two or more participants display a transient inter-dependence on many levels (Shockley et al., 2009; Fusaroli et al., 2014). This third-person account lends itself well to ethological and experimental observation and modeling. A well-worked mathematical framework for describing how autonomous systems that interact in real time can give rise to emergent phenomena at the collective level is available, e.g., as illustrated by the field of coordination dynamics (Kelso, 1995; Oullier and Kelso, 2009). Social cognitive neuroscience has recently begun to recognize that nervous systems of interacting individuals behave quite differently from those of solitary subjects, and often become inter-dependent (Hari and Kujala, 2009; Babiloni and Astolfi, 2012; Schilbach et al., 2013). This opens up a vast empirical research agenda for the future.

But the shifting ground of subjectivity that is here espoused poses challenges for description from a phenomenological or experiential point of view. Here, the recent concept of participatory sense-making may be of assistance (De Jaegher and Di Paolo, 2007; Fuchs and De Jaegher, 2009). Participatory sense-making extrapolates from the basic enactive account that grounds sense-making (perception/action in the service of the generation of meaning) in the adaptive interaction of an autonomous agent with its environment (Froese and Di Paolo, 2011). Building on this perspective, participatory sense-making describes how the moment-to-moment interaction of two subjects gives rise to a mutuality in their joint sense-making, allowing for the joint creation of meaning. On this account, the emergent domain constituted by the inter-dependent activities of two or more subjects warrants treatment as a phenomenological domain in its own right (Cummins, 2013b). Intersubjectivity then is the enactment of a novel phenomenological domain in the sustained, real time coordinated activities of two or more people. There appears to be a convergence of the theoretical vocabulary and the demands raised by empirical studies that bodes well for further scientific work.

A host of open questions relate to the role of clock time and synchronized behavior. In collective speaking, we observe highly coordinated action that relies, not on a common external beat or timekeeper, but on shared knowledge among interactants. Highly synchronized behavior that is scaffolded by an external beat is also very common, as in music making, marching, or dancing, but this kind of collective entrainment does not seem to bring with it an automatic sense of commitment to underlying beliefs or intentions. We are all familiar with western school kids dancing happily to the religiously tinged beats of Bob Marley, without worrying about whether they really subscribe to the tenets of Rastafarianism. Much work remains to be done in gaining a better understanding of how collective coordinated behavior gives rise to collective intentionality, and what the necessary preconditions for that in the contributing individuals are.

A willingness to countenance subjective poles that are not co-extensive with the individual person, and that rise and fade in a dynamic fashion, is incompatible with the grounding assumptions of much of conventional psychology. Of course, psychology itself has grappled since its inception with the boundaries of the subject (Dewey, 1896). One way of describing the subject matter of psychology is with reference to the twin poles of experience and behavior, for which a causal account is sought. This approach looks out at the world from a subject whose existence, persistence, and integrity is taken for granted. The approach taken here, and enabled by the enactive framework more generally, is to reverse the direction of inquiry, from a view toward experience (whose?) and behavior (by whom?), and to look instead at the shifting referents of the personal pronouns “I,” “we,” “you,” etc. It is here that it becomes apparent that the received view of language will not serve, any more than the notion of a solipsistic mind. Of course the contemporary scientific view of language is deeply rooted in a specific set of psychological commitments, and a view of mind as information processing, that together gave birth to the cognitivist worldview. Adopting a different stance with respect to the ground of experience must, it seems, go hand in hand with a willingness to question the boundaries that have traditionally served to demarcate the linguistic domain. This opens up the enticing prospect that we might begin to question, negotiate, and re-evaluate just what, and who, “we” think “we” are.

In Seeger (2004), an account is provided of the way music and song are integrated into the lives of the Suyá people of the Amazon basin. Some songs, the shout songs, are sung from what we might consider a conventional egocentric perspective. Others are sung in unison. Of these Seeger notes:

The Suyá men said they sang shout songs for their sisters… When I asked them for whom they sang unison songs, they responded that they simply sang them. They weren't for anyone. A man did not sing a unison song as a brother, lover, or individual. He sang it as a member of a group, whose identity was partly established through the song. Thus, they sang for a general audience: the act of singing was the statement. In some sense, invocations had no audience at all… Seeger, 2004, p. 83).

## Acknowledgment

I am indebted to three anonymous reviewers who provided very thoughtful feedback which improved the present contribution.

### Conflict of interest statement

The author declares that the research was conducted in the absence of any commercial or financial relationships that could be construed as a potential conflict of interest.

## Footnotes

^1^A complementary account of languaging from an enactive perspective is provided in Bottineau (2010). This account adheres to a more conventional view of what the domain of language is than adopted here, but many of the fundamental concerns raised therein resonate with the themes of this article.

^2^The famous Latin phrase does not appear in the Second Meditation, where the original argument is most clearly made.

^3^My thanks to the anonymous reviewer who pointed out that the stark dichotomy between spoken and written texts has become considerably more complex.

^4^I hypothesize—I am not sure how one might measure relative frequency here.

^5^Small wonder then that the appearance of “language” appears utterly mysterious from the vantage point of modern linguistics (Hauser et al., 2014). The discipline has defined its own subject almost out of existence.

## References

[B1] Aristotle. (1986). De Anima (On The Soul). London: Penguin

[B2] Austin J. L. (1975). How to do Things with Words. Oxford: Oxford University Press 10.1093/acprof:oso/9780198245537.001.0001

[B3] Auvray M. Lenay C. Stewart J. (2009). Perceptual interactions in a minimalist virtual environment. New Ideas Psychol. 27, 32–47 10.1016/j.newideapsych.2007.12.002

[B4] Babiloni F. Astolfi L. (2012). Social neuroscience and hyperscanning techniques: past, present and future. Neurosci. Biobehav. Rev. [Epub ahead of print]. 10.1016/j.neubiorev.2012.07.006 22917915 PMC3522775

[B5] Bell C. (1988). Ritualization of texts and textualization of ritual in the codification of Taoist liturgy. Hist. Relig. 27, 366–392 10.1086/463128

[B6] Bottineau D. (2010). Language and enaction in Enaction: Toward a New Paradigm for Cognitive Science, eds Stewart J. R. Gapenne O. Di Paolo E. A. (Cambridge, MA: MIT Press), 267

[B7] Campbell N. (2007). On the use of nonverbal speech sounds in human communication. Verbal Nonverbal Commun. Behav. 4775, 117–128 10.1007/978-3-540-76442-7_11

[B8] Carr D. (ed.). (1987). Cogitamus ergo sumus: the intentionality of the first-person plural, in Interpreting Husserl (Cambridge, UK: Springer), 281–296 10.1007/978-94-009-3595-2_15

[B9] Chanan M. (1995). Repeated Takes: A Short History of Recording and its Effects on Music. New York, NY: Verso

[B10] Clark H. H. Brennan S. E. (1991). Grounding in communication, in Perspectives on Socially Shared Cognition, ed Rogoff B. (Washington, DC: American Psychological Association), 127–149 10.1037/10096-006

[B11] Connor S. (2000). Dumbstruck: A Cultural History of Ventriloquism. Oxford: Oxford University Press 10.1093/acprof:oso/9780198184331.001.0001

[B12] Cowley S. J. Love N. (2006). Language and cognition, or, how to avoid the conduit metaphor, in Bridges and Walls in Metalinguistic Discourse (Frankfurt: Peter Lang), 135–154

[B13] Cummins F. (2003). Practice and performance in speech produced synchronously. J. Phon. 31, 139–148 10.1016/S0095-4470(02)00082-7

[B14] Cummins F. (2009). Rhythm as entrainment: the case of synchronous speech. J. Phon. 37, 16–28 10.1016/j.wocn.2008.08.003

[B15] Cummins F. (2012). Gaze and blinking in dyadic conversation: a study in coordinated behaviour among individuals. Lang. Cogn. Process. 27, 1525–1549 10.1080/01690965.2011.615220

[B16] Cummins F. (2013a). Joint speech: the missing link between speech and music? Percept. Rev. Cogn. Music. 1, 17–32 Available online at: http://www.abcogmus.org/journals/index.php/percepta/article/view/16/30

[B17] Cummins F. (2013b). Towards an enactive account of action: speaking and joint speaking as exemplary domains. Adapt. Behav. 21, 178–186 10.1177/1059712313483144

[B18] Cummins F. (2014). The remarkable unremarkableness of joint speech, in Proceedings of the 10th International Seminar on Speech Production (Cologne), 73–77

[B19] Cummins F. Li C. Wang B. (2013). Coupling among speakers during synchronous speaking in English and Mandarin. J. Phon. 41, 432–441 10.1016/j.wocn.2013.07.001

[B20] Dale R. Fusaroli R. Duran N. Richardson D. C. (2013). The self-organization of human interaction. Psychol. Learn. Motiv. 59, 43–95 10.1016/B978-0-12-407187-2.00002-2

[B21] Daston L. J. Galison P. (2007). Objectivity. New York, NY: Zone Books

[B22] De Jaegher H. Di Paolo E. (2007). Participatory sense-making: an enactive approach to social cognition. Phenomenol. Cogn. Sci. 6, 485–507 10.1007/s11097-007-9076-923532205

[B23] Dellwo V. Friedrichs D. (2012). Variability of speech rhythm in synchronous speech, in Proceedings of Speech Prosody 2012, Vol. 2 (Chicago, IL), 539–542

[B24] Deutsch D. Henthorn T. Lapidis R. (2011). Illusory transformation from speech to song. J. Acoust. Soc. Am. 129, 2245–2252 10.1121/1.3562174 21476679

[B25] Dewey J. (1896). The reflex arc concept in psychology. Psychol. Rev. 3, 357 10.1037/h0070405

[B26] Everett D. L. (2009a). Don't Sleep, There are Snakes: Life and Language in the Amazonian Jungle. New York, NY: Random House LLC

[B27] Everett D. L. (2009b). Pirahã culture and grammar: a response to some criticisms. Language 85, 405–442 10.1353/lan.0.0104

[B28] Fodor J. A. (1975). The Language of Thought. Cambridge, MA: Harvard University Press

[B29] Froese T. Di Paolo E. (2011). The enactive approach: theoretical sketches from cell to society. Pragmat. Cogn. 19, 1–36 10.1075/pc.19.1.01fro

[B30] Froese T. Iizuka H. Ikegami T. (2014). Embodied social interaction constitutes social cognition in pairs of humans: a minimalist virtual reality experiment. Sci. Rep. 4:3672 10.1038/srep03672 24419102 PMC3890942

[B31] Fuchs T. De Jaegher H. (2009). Enactive intersubjectivity: participatory sense-making and mutual incorporation. Phenomenol. Cogn. Sci. 8, 465–486 10.1007/s11097-009-9136-4

[B32] Fusaroli R. Rączaszek-Leonardi J. Tylén K. (2014). Dialog as interpersonal synergy. New Ideas Psychol. 32, 147–157 10.1016/j.newideapsych.2013.03.005

[B33] Fusaroli R. Tylén K. (2012). Carving language for social coordination: a dynamical approach. Interact. Stud. 13, 103–124 10.1075/is.13.1.07fus

[B34] Hari R. Kujala M. V. (2009). Brain basis of human social interaction: from concepts to brain imaging. Physiol. Rev. 89, 453–479 10.1152/physrev.00041.2007 19342612

[B35] Hauser M. D. Chomsky N. Tecumseh Fitch W. (2002). The faculty of language: what it is. who has it, and how did it evolve? Science 298, 1569–1579 10.1126/science.298.5598.1569 12446899

[B36] Hauser M. D. Yang C. Berwick R. C. Tattersall I. Ryan M. Watumull J. (2014). The mystery of language evolution. Front. Psychol. Lang. Sci. 5:401 10.3389/fpsyg.2014.00401 24847300 PMC4019876

[B37] Huang C.-T. J. (1984). On the distribution and reference of empty pronouns. Linguist. Inquiry 15, 531–574

[B38] Hutto D. D. Myin E. (2013). Radicalizing Enactivism: Basic Minds Without Content. Cambridge, MA: MIT Press

[B39] Kelso J. A. S. (1995). Dynamic Patterns. Cambridge, MA: MIT Press

[B40] Kim M. Nam H. (2008). Synchronous speech and speech rate. J. Acoust. Soc. Am. 123, 3736 10.1121/1.2935251

[B41] Koriat A. (2012). When are two heads better than one and why? Science 336, 360–362 10.1126/science.1216549 22517862

[B42] Krivokapić J. (2007). Prosodic planning: effects of phrasal length and complexity on pause duration. J. Phon. 35, 162–179 10.1016/j.wocn.2006.04.001 18379639 PMC2131696

[B43] Latash M. (2008). Synergy. Oxford: Oxford University Press 10.1093/acprof:oso/9780195333169.001.0001

[B44] Latour B. (2013). An Inquiry into Modes of Existence. Cambridge, MA: Harvard University Press

[B45] Linell P. (2005). Written Language Bias in Linguistics. New York, NY: Routledge 10.4324/9780203342763

[B46] McLuhan M. (1964). Understanding Media: The Extensions of Man. New York, NY: McGraw-Hill

[B47] Michaels A. Mishra A. Dolce L. Raz G. Triplett K. (2010). Grammars and Morphologies of Ritual Practices in Asia. Wiesbaden, DE: Harrassowitz Verlag

[B48] Murray L. Trevarthen C. (1986). The infant's role in mother-infant communications. J. Child Lang. 13, 15–29 10.1017/S0305000900000271 3949895

[B49] O'Dell M. Nieminen T. Mustanoja L. (2010). Assessing rhythmic differences with synchronous speech, in Speech Prosody 2010-Fifth International Conference, Vol. 100141 (Hong Kong), 1–4

[B50] Okamoto-Barth S. Call J. Tomasello M. (2007). Great apes' understanding of other individuals' line of sight. Psychol. Sci. 18, 462–468 10.1111/j.1467-9280.2007.01922.x 17576288

[B51] Olson D. R. (1996). The World on Paper. Cambridge, UK: Cambridge University Press

[B52] Ong W. (1982). Orality and Literacy: The Technologizing of the Word. London: Methuen & Co 10.4324/9780203328064

[B53] Oullier O. Kelso J. A. S. (2009). Social coordination, from the perspective of coordination dynamics, in Encyclopedia of Complexity and Systems Science, ed Meyers R. A. (Heidelberg: Springer), 8198–8213 10.1007/978-0-387-30440-3_486

[B54] Pickering M. J. Garrod S. (2004). Toward a mechanistic psychology of dialogue. Behav. Brain Sci. 27, 169–190 10.1017/S0140525X04000056 15595235

[B55] Pickering M. J. Garrod S. (2014). Self-, other-, and joint monitoring using forward models. Front. Hum. Neurosci. 8:132 10.3389/fnhum.2014.00132 24723869 PMC3971194

[B56] Pinker S. (1999). How the mind works. Ann. N.Y. Acad. Sci. 882, 119–127 10.1111/j.1749-6632.1999.tb08538.x 10415890

[B57] Richardson D. C. Dale R. Kirkham N. Z. (2007). The art of conversation is coordination: common ground and the coupling of eye movements during dialogue. Psychol. Sci. 18, 407–413 10.1111/j.1467-9280.2007.01914.x 17576280

[B58] Riley M. A. Richardson M. J. Shockley K. Ramenzoni V. C. (2011). Interpersonal synergies. Front. Psychol. 2:38 10.3389/fpsyg.2011.00038 21716606 PMC3110940

[B59] Ryle G. (1949). The Concept of Mind. New York, NY: Barnes & Noble

[B60] Saussure F. D. (1959/1916). Course in General Linguistics. New York, NY: Philosophical Library

[B61] Schegloff E. A. (1991). Conversation analysis and socially shared cognition, in Socially Shared Cognition, eds Resnick L. B. Levine J. Behrend S. D. (Washington, DC: American Psychological Association), 150–171

[B62] Schilbach L. Timmermans B. Reddy V. Costall A. Bente G. Schlicht T. (2013). Toward a second-person neuroscience. Behav. Brain Sci. 36, 393–414 10.1017/S0140525X12000660 23883742

[B63] Seeger A. (2004). Why Suyá sing: A Musical Anthropology of an Amazonian People. Chicago, IL: University of Illinois Press

[B64] Sheets-Johnstone M. (1999). Emotion and movement. a beginning empirical-phenomenological analysis of their relationship. J. Conscious. Stud. 6, 11–12

[B65] Shockley K. Richardson D. C. Dale R. (2009). Conversation and coordinative structures. Topics Cogn. Sci. 1, 305–319 10.1111/j.1756-8765.2009.01021.x 25164935

[B66] Stewart J. (2010). Foundational issues in enaction as a paradigm for cognitive science: from the origin of life to consciousness and writing, in Enaction: Toward a New Paradigm for Cognitive Science, eds Stewart J. R. Gapenne O. Di Paolo E. A. (Cambridge, MA: MIT Press), 1–31

[B67] Thibault P. J. (2011). First-order languaging dynamics and second-order language: the distributed language view. Ecol. Psychol. 23, 210–245 10.1080/10407413.2011.591274

[B68] Tomasello M. Carpenter M. Call J. Behne T. Moll H. (2005). Understanding and sharing intentions: the origins of cultural cognition. Behav. Brain Sci. 28, 675–691 10.1017/S0140525X05000129 16262930

[B69] Tomasello M. Farrar M. J. (1986). Joint attention and early language. Child Dev. 57, 1454–1463 10.2307/1130423 3802971

[B70] Tomasello M. Hare B. Lehmann H. Call J. (2007). Reliance on head versus eyes in the gaze following of great apes and human infants: the cooperative eye hypothesis. J. Hum. Evol. 52, 314–320 10.1016/j.jhevol.2006.10.001 17140637

[B71] Volta Laboratory (2013). New Optical Scan Results From The Smithsonian Volta Laboratory Collection. Catalogue No. 312123. Available online at: http://bio16p.lbl.gov/volta-release-2013.html

[B72] Vygotsky L. (1986). Thought and Language. Cambridge, MA: MIT Press

[B73] Wagner P. Malisz Z. Kopp S. (2014). Gesture and speech in interaction: an overview. Speech Commun. 57, 209–232 10.1016/j.specom.2013.09.008

